# Research overview—2025: integrating scientific progress to accelerate global tuberculosis elimination

**DOI:** 10.3389/ftubr.2026.1805962

**Published:** 2026-05-25

**Authors:** Patrick K. Moonan, Timothy H. Holtz

**Affiliations:** 1 U.S. Centers for Disease Control and Prevention, Division of Global HIV and Tuberculosis, Atlanta, GA, United States,; 2 Sumner M. Redstone Global Center for Prevention and Wellness, The George Washington University Milken Institute School of Public Health, Washington, DC, United States

**Keywords:** antituberculous treatment, diagnostics, drug development, research, tuberculosis, vaccine development

## Abstract

Major scientific advances across the tuberculosis research continuum emerged in 2025, offering new opportunities to accelerate progress toward global elimination. This review synthesizes developments in diagnosis, treatment, prevention, and post-disease care, highlighting innovations that broaden access, strengthen mechanistic insight, and expand the scope of actionable tools. Diagnostic progress included artificial intelligence–enabled chest imaging, noninvasive molecular testing using tongue and stool samples, simple stool-based procedures suitable for decentralized settings, biomarkers that support treatment monitoring, low-complexity nucleic acid tests for extrapulmonary disease, and new point-of-care methods for detecting infection. Culture-free sequencing approaches further improved rapid identification of drug resistance. Therapeutic advances featured shorter all-oral regimens for drug-susceptible and rifampin-resistant disease, along with emerging compounds that target essential microbial pathways. Additional progress in host-directed therapies, vaccines, and nanoparticle-based drug delivery broadened preventive and therapeutic options. Conceptual developments challenged the validity of symptom-based disease categories and supported biologically grounded terminology, while consensus definitions for post-tuberculosis lung disease clarified the substantial long-term respiratory burden among survivors. Despite this momentum, health-system limitations and inequitable access continue to constrain real-world impact. Collectively, the scientific advances of 2025 were substantial and outline a clearer mechanistic and programmatic path toward tuberculosis elimination, but their success will depend on effective implementation and integration into routine care.

## 1 Introduction

Tuberculosis (TB) remains one of the leading causes of death from a single infectious agent worldwide (1). Few high-burden countries are on track to achieve the 2030 End TB Strategy milestones, which call for a 90% reduction in incidence and a 95% reduction in deaths compared with 2015 levels (2, 3). Global TB incidence is declining at only 1.5%–2% annually—well below the trajectory required to meet 2030 goals—and mortality reductions remain insufficient, especially among people living with HIV, children, and marginalized populations (1, 2). These epidemiologic trends reflect longstanding structural constraints, including delayed diagnosis, gaps in linkage to and completion of treatment, persistent limitations in drug-resistant TB care, weak supply chains, and chronic underfinancing of national TB programs (3, 4).

Against this backdrop of limited epidemiologic progress, 2025 produced notable scientific advances across the TB continuum. Progress in diagnostics, therapeutics, vaccines, and immunology coincided with renewed attention to post-TB morbidity and the language used to describe disease states. However, new tools alone are insufficient to alter the global TB trajectory. Health-system limitations, regulatory delays, and insufficient access to diagnostics and treatment represent persistent barriers to implementation (1–4). Without clear strategies to bridge the gap between discovery and delivery, scientific breakthroughs may fail to generate population-level impact. Recent work highlights that key questions in tuberculosis remain unresolved, including how to define the disease spectrum, identify appropriate vaccine endpoints, and address limitations of symptom-based screening that may miss early or subclinical disease (5). These ongoing uncertainties shape research priorities and implementation strategies discussed in this review.

This report synthesizes these developments using a mechanistic framework to contextualize therapeutic advances, and considers how financing and implementation realities may influence their impact that will shape progress toward the 2030 elimination goals. Briefly, we conducted a structured PubMed search using the term “tuberc*” restricted to January 1–December 31, 2025, which yielded 12,351 records. This review is intended as a narrative synthesis rather than a formal systematic review and is guided by considerations of scientific rigor, novelty, translational relevance, and policy impact. Accordingly, we prioritized randomized trials, systematic reviews, high-impact mechanistic studies, and major policy updates, with selective inclusion of late-phase preprints when programmatically relevant. Although this discussion focuses on publications from 2025, evidence that has already informed global recommendations or is being implemented programmatically is included to contextualize the evolving evidence base supporting current TB prevention and treatment strategies.

## 2 Diagnostic advances in 2025

Diagnostic innovation remains essential for accelerating progress toward TB elimination, particularly as millions of cases continue to go undiagnosed each year (1). In 2025, several advances substantially improved the accessibility, speed, and reliability of TB testing across diverse clinical and programmatic settings.

Artificial intelligence–assisted chest radiography improved diagnostic sensitivity and reader consistency, reducing time to triage and standardizing interpretation in high-volume programs across India, Eastern Europe, and Southeast Asia (6). Garg et al. found that ultraportable chest radiography with AI interpretation was a cost-effective screening and triage approach for active tuberculosis case finding in Nigeria, improving diagnostic yield while reducing reliance on expert readers (7). In a systematic review of 152 studies, AI-based methods—particularly deep learning applied to radiographic data—demonstrated consistently high diagnostic performance for tuberculosis detection in many settings, with pooled sensitivity estimates commonly exceeding 85%, depending on study design and reference standard (8). WHO subsequently recognized computer-aided detection tools as critical for lowering the proportion of undetected disease (9).

Methods to use non-invasive specimen collection for molecular assay testing also advanced. Tongue-swab polymerase-chain-reaction (PCR)–based tests demonstrated diagnostic accuracy comparable to sputum-based GeneXpert MTB/RIF^®^ (Cepheid, Sunnyvale, CA) when evaluated against the same reference standards (10), and quantitative approaches such as TB-EASY showed high performance across bacterial-load strata (11). The SMART4TB multi-country diagnostic accuracy study of 1,168 participants with presumptive tuberculosis in the Philippines, South Africa, Nigeria, and Zambia found that tongue-swab–based testing achieved high specificity (99.6%) and moderate sensitivity (66.0%) against a culture-based reference standard, with low non-actionable result rates at most sites (12). These characteristics support its use for individuals unable to produce sputum—including children, older adults, and people living with HIV. Programmatically, the moderate sensitivity of tongue-swab and stool-based assays—particularly in adults—supports their use within diagnostic algorithms rather than as standalone tests for treatment initiation, with confirmatory testing remaining essential for adult clinical decision-making.

Simple One-Step (SOS) stool-based molecular testing made notable progress. Optimization studies of SOS GeneXpert Ultra showed that real-world variation in stool quantity, transport time, and ambient temperature did not meaningfully affect error rates or positivity, reinforcing suitability for decentralized, child-friendly testing (13, 14). Programmatic evaluations demonstrated high feasibility and acceptability among health workers and increased bacteriologic confirmation of pediatric TB when stool testing complemented respiratory sampling (15). Beyond diagnosis, stool-based testing emerged as a potential tool for treatment monitoring. A multi-country study found that stool *Mycobacterium tuberculosis* quantification correlated closely with sputum culture, provided results more rapidly, and used a more accessible specimen, while also identifying individuals at elevated risk for drug resistance and persistent two-month culture positivity; however, no single biomarker reliably predicted symptom resolution or mortality (16). Stool-based molecular testing has improved access to pediatric TB diagnosis but has modest standalone yield in paucibacillary disease. Accordingly, WHO recommends treatment decision algorithms (TDAs) for children under 10 years with presumptive TB (17). Recent analyses show TDAs have high sensitivity but suboptimal specificity and are cost-effective in high-risk groups, such as children with severe acute malnutrition, supporting their use within integrated diagnostic approaches (18).

WHO guidance issued early in 2025 emphasized concurrent testing using multiple specimen types in children, including parallel collection of stool and respiratory samples, with urine lipoarabinomannan (LAM) testing recommended for children living with HIV, to improve microbiologic confirmation and reduce diagnostic delay (17, 19, 20). This approach reflects accumulating evidence that no single specimen type performs optimally across pediatric sub-populations and that combined sampling can increase diagnostic yield when incorporated within structured diagnostic algorithms.

A Cochrane meta-analysis evaluating low-complexity automated nucleic acid amplification tests for extrapulmonary tuberculosis demonstrated high specificity and moderate-to-high sensitivity for TB detection and rifampicin resistance across common specimen types, including lymph node aspirates, cerebrospinal fluid, pleural fluid, and other extrapulmonary samples (21). This evidence directly informed WHO’s 2025 endorsement of these platforms for decentralized use (17).

There was also progress to improve the diagnosis of TB infection. In a large cohort of healthcare workers in Sevagram, India, the TB antigen-based skin test SIILTIBCY^®^ (CY-TB) demonstrated high specificity (97%) and acceptable sensitivity (76%) compared with QuantiFERON-TB Gold Plus^®^, with substantial agreement (*κ* = 0.77) across 1,338 paired results (22). Complementary cost-effectiveness analyses suggested that CYTB may be economically advantageous, particularly under reduced pricing scenarios, supporting its scalability (23). In parallel, point-of-care blood-based assays advanced. A cross-sectional study of household contacts in Vietnam showed that the STANDARD F TB-Feron FIA assay achieved high sensitivity (~88%) and strong concordance (~92%) with QuantiFERON-TB Gold Plus^®^, while providing same-day digital readouts without centralized laboratory infrastructure (24). Together, these tests broaden the landscape of TB infection diagnostics and strengthen the feasibility of streamlined test-and-treat strategies.

Diagnostic algorithms for high-risk groups were further refined. Among migrants, combining interferon-*γ* release assays, chest radiography, and GeneXpert Ultra improved diagnostic yield compared with single-test strategies (25). In children, parallel sampling of gastric aspirates, induced sputum, and nasopharyngeal specimens increased microbiologic confirmation (19). Among adults living with HIV, integrating urine LAM-based testing with AI-assisted chest radiography more than doubled inpatient TB treatment initiation (20).

Additional adjunct and emerging diagnostic approaches underwent early clinical evaluation in 2025. Specimen pooling strategies for molecular testing were explored in research and pilot implementation settings as potential means of improving laboratory efficiency and conserving resources in high-volume screening contexts, although performance remains sensitive to bacillary load, pool size, and disease prevalence (8, 9). Urine LAM-based testing continued to be evaluated beyond advanced HIV disease within risk-stratified diagnostic algorithms (17, 20). Breath-based volatile organic compound (VOC) signatures advanced through early clinical validation as noninvasive screening approaches, with their role in triage and population-level screening remaining under investigation (8). Point-of-care ultrasound was further evaluated for extrapulmonary and pediatric TB, particularly for lymphadenopathy, pleural disease, and abdominal involvement, where it may complement microbiologic testing in selected settings (17, 21).

Molecular innovation also extended to urine cell-free DNA (cfDNA) assays, which demonstrated early analytical and clinical feasibility for TB detection, with potential applications to treatment monitoring requiring further validation (26). In parallel, CRISPR-based molecular diagnostics, including assays performed directly on sputum, demonstrated high analytic sensitivity in early-phase and proof-of-concept studies, supporting continued clinical evaluation (27). While these platforms are not yet suitable for routine programmatic use, they illustrate the expanding range of molecular approaches under investigation.

Targeted next-generation sequencing matured into a practical tool for rapid drug-resistance profiling. Multi-country studies reported high rates of successful resistance profiling directly from primary specimens, with strong concordance to phenotypic methods for several first- and second-line drugs, including rifampicin and isoniazid, and emerging evidence for newer agents (26). Although culture-free sequencing offers major gains in speed and resistance detection, operational barriers—including cost, turnaround time, contamination control, and bioinformatics capacity—currently limit routine programmatic use.

Collectively, these technological and operational advances could expand the diagnostic ecosystem, enabling earlier case identification, more effective treatment monitoring, and improved access to high-quality testing for populations historically underserved by conventional approaches.

## 3 Mechanistic landscape of tuberculosis drug targets

A mechanistic understanding of tuberculosis therapeutics is essential for interpreting the advances reported in 2025. As summarized by Edwards and Field (27), antimycobacterial drugs act on three principal domains of *M. tuberculosis* physiology: cell-wall biosynthesis; energy metabolism and oxidative phosphorylation; protein or nucleic acid synthesis; with additional contributions from efflux-pump regulation (Table 1).

The mycobacterial cell wall is a highly structured, multilayered barrier composed of a peptidoglycan scaffold, an arabinogalactan polymer, and an outer membrane enriched with mycolic acids (28). Multiple drug classes disrupt this architecture. Isoniazid and ethionamide inhibit the *inh*A enzyme required for mycolic-acid synthesis. Ethambutol blocks arabinan polymerization by inhibiting arabinosyl transferases. Benzothiazinones—including BTZ-043 and PBTZ-169—target DprE1, an essential epimerase in arabinogalactan synthesis, while carbapenems, especially when combined with *β*-lactamase inhibitors, inhibit D,D-transpeptidases critical for peptidoglycan crosslinking.

Energy metabolism represents a second major therapeutic target. Bedaquiline and next-generation diarylquinolines inhibit adenosine triphosphate (ATP) synthase by binding its c-subunit, collapsing intracellular ATP stores (28). In parallel, cytochrome bc1 inhibitors such as telacebec (Q203) block the QcrB subunit of the respiratory chain, disrupting oxidative phosphorylation (28, 29). Because both ATP production and electron transport are essential under replicating and metabolically constrained conditions, these agents hold strong potential for regimen shortening.

The third domain includes inhibitors of protein and nucleic acid synthesis. Oxazolidinones (i.e., linezolid, tedizolid, sutezolid, delpazolid, and TBI-223) inhibit mycobacterial protein synthesis by binding to the peptidyl transferase center of the 50S ribosomal subunit (23S rRNA), thereby blocking peptide bond formation during elongation (30). Fluoroquinolones and novel gyrase inhibitors disrupt deoxyribonucleic acid (DNA) replication (31), while leucyl-tRNA synthetase inhibitors such as oxaboroles (e.g., GSK3036656; ganfeborole) impair aminoacylation and protein synthesis (32).

Efflux mechanisms also influence drug activity and resistance. Mutations in *Rv*0678 upregulate the MmpL5–MmpS5 efflux system, reducing susceptibility to diarylquinolines and riminophenazines (33). Although efflux-pump inhibitors like verapamil show synergistic effects in experimental systems, their clinical utility remains unproven.

Together, these mechanistic insights provide a foundation for interpreting the therapeutic and pipeline advances that follow, particularly efforts to design regimens that combine complementary modes of action to maximize bactericidal and sterilizing activity.

## 4 Therapeutic advances in 2025

Therapeutic innovation remains central to achieving the End TB Strategy milestones, as treatment success, regimen tolerability, and duration directly shape TB incidence and mortality. In 2025, several clinical studies advanced the landscape for both drug-susceptible and drug-resistant antituberculosis treatment (ATT).

Randomized clinical trials have established the effectiveness of a fully oral four-month regimen for drug-susceptible TB—consisting of rifapentine, moxifloxacin, isoniazid, and pyrazinamide—with Study 31 demonstrating noninferiority to the traditional six-month standard (34, 35). More recent analyses provide additional nuance: a 2025 subgroup analysis among participants with diabetes reported favorable efficacy and safety, with the rifapentine–moxifloxacin arm showing the lowest proportion of unfavorable outcomes.

Despite this efficacy, emerging programmatic data highlight important implementation challenges. Reports from U.S. settings, including New York City, indicate that a substantial proportion of patients are not eligible for the shortened regimen, and among those who initiate treatment, interruption rates can be high (36). Notably, grade 2 adverse events—while not dose-limiting in the original trial—have contributed to treatment discontinuation in real-world practice (36). These findings underscore the gap between trial efficacy and implementation feasibility, reinforcing the need for careful patient selection and strengthened pharmacovigilance as the regimen is scaled.

Mechanistically, rifapentine and moxifloxacin provide potent bactericidal and sterilizing activity through inhibition of mycobacterial RNA polymerase and DNA gyrase, while pyrazinamide retains unique activity against bacilli in acidic intracellular environments (34). Together, these complementary actions enhance early bacterial killing and reduce relapse risk.

For rifampin-resistant and multidrug-resistant tuberculosis (MDR tuberculosis), a large multinational Phase 3 trial evaluated five all-oral regimens lasting six to nine months (37). Four regimens were noninferior to existing standards and achieved favorable outcomes of approximately 80%, with improved tolerability compared with injectable-containing regimens. These regimens deliberately combine mechanistically orthogonal agents—including ATP synthase inhibition (e.g., bedaquiline), protein-synthesis inhibition (e.g., linezolid), and DNA gyrase inhibition (e.g., fluoroquinolones)—to maximize sterilization across bacterial subpopulations (37). Fluoroquinolone-resistant drug-resistant tuberculosis, now the most common pre-XDR phenotype, has been directly addressed in recent regimen evaluations, most notably the END-TB-Q Phase 3 trial (37). Notably, in prespecified subgroup analyses of the End TB regimens trial, delamanid, moxifloxacin, clofazimine, pyrazinamide (DMCZ) regimen demonstrated strong effectiveness against bedaquiline-resistant tuberculosis (86%) with a low adverse-event rate (18%), exceeding outcomes observed with control and comparator regimens and underscoring its potential utility in the setting of emerging diarylquinoline resistance (37). The END-TB-Q and BEAT-TB approaches both employed bedaquiline-based regimens with systematic fluoroquinolone drug-susceptibility testing to guide regimen selection; in BEAT-TB, an initial bedaquiline–delamanid–linezolid–levofloxacin–clofazimine backbone was subsequently tailored by discontinuing clofazimine for fluoroquinolone-susceptible disease or levofloxacin for fluoroquinolone-resistant disease, as described in programmatic and guideline sources pending availability of a primary trial publication (38). Shorter treatment duration and elimination of injectables represent major advances toward the End TB targets by improving adherence and reducing health-system burden. The End-TB Phase 3 randomized trial demonstrated the efficacy of shortened, all-oral regimens for rifampin-resistant tuberculosis, providing key evidence to support regimen simplification and improved tolerability. These findings directly informed future guideline updates, which endorsed shorter treatment options, including nine-month regimens and a six-month bedaquiline-based combination for eligible patients (39). This shift represents a major advance in aligning clinical trial evidence with global policy to improve treatment outcomes and programmatic feasibility.

Treatment of pediatric rifampin-resistant and MDR tuberculosis also improved. A large individual participant data meta-analysis demonstrated substantially higher success in children receiving regimens containing two or more WHO Group A drugs—most commonly bedaquiline, linezolid, and a fluoroquinolone (40). Mechanistically, simultaneous disruption of ATP synthesis, protein synthesis, and DNA replication yields broad activity across physiologic states, underscoring the urgent need for child-friendly formulations and improved access to high-potency regimens.

Updated clinical practice guidelines released by the American Thoracic Society, Centers for Disease Control and Prevention, European Respiratory Society, and Infectious Diseases Society of America incorporated these findings by formally endorsing four-month regimens for drug-susceptible TB and fully oral, shortened regimens for rifampin-resistant TB (41). These updates signal broad consensus on the feasibility and safety of shortened regimens and will shape national policy adoption throughout the decade.

### 4.1 Preventing tuberculosis following exposure to multidrug-resistant disease

Because MDR tuberculosis requires a complex and prolonged treatment, preventing progression among exposed contacts is a critical control strategy. In 2025, two randomized controlled trials evaluated fluoroquinolone-based tuberculosis preventive treatment (TPT) for household contacts of patients with MDR tuberculosis (42, 43).

The VQUIN trial in Vietnam randomized 2,041 household contacts with TB infection to receive six months of daily oral levofloxacin-based TPT or placebo, providing robust evidence that strengthened the scientific basis for existing global recommendations on TPT for contacts of drug-resistant TB (42). At 30 months, tuberculosis incidence was 0.6% in the levofloxacin group compared with 1.1% in the placebo group. The TB-CHAMP trial in South Africa randomized 922 children exposed to MDR tuberculosis to either levofloxacin-based TPT or placebo (43). Neither trial reached statistical significance individually due to lower-than-expected incidence.

A preplanned individual participant data meta-analysis demonstrated a 60% relative reduction in tuberculosis incidence among adult and pediatric MDR tuberculosis contacts receiving levofloxacin, although musculoskeletal adverse events were more frequent among participants aged 10 years and older (44). Several ongoing trials—including PHOENIx, evaluating six months of delamanid (45), and BREACH-TB, evaluating six months of bedaquiline (46)—are expected to further inform TPT strategies for contacts exposed to fluoroquinolone-resistant drug-resistant tuberculosis once outcome data become available. These findings highlight both the promise and limitations of chemoprevention and underscore the complementary role of vaccines and immune-based strategies, discussed below, in achieving durable protection against tuberculosis.

## 5 Compound candidates and novel drugs evaluated in 2025

Beyond regimen optimization, 2025 saw notable progress in mechanistically novel compounds that diversify and strengthen the TB therapeutic pipeline. Developed largely through the TB Alliance and global partnerships (47), these agents expand key biochemical lineages targeting ATP synthase, cytochrome bc1, and DprE1—targets with strong potential to shorten therapy, overcome existing resistance (48, 49), and improve tolerability.

### 5.1 ATP synthase inhibitors: TBAJ-876 (sorfequiline) and TBAJ-587

ATP synthase remains one of the most validated and attractive targets for TB drug development (28). Bedaquiline revolutionized multidrug-resistant TB treatment by collapsing ATP production, killing bacilli across metabolic states, and enabling shorter all-oral regimens. Next-generation diarylquinolines aim to enhance potency, safety, and pharmacokinetics.

TBAJ-876 (i.e., sorfequiline) is the most advanced of these candidates. Engineered for improved binding affinity to the ATP synthase c-subunit, it provides stronger inhibition of oxidative phosphorylation than bedaquiline (28, 50). In the multi-country Phase 2 NC-009 trial (51), a regimen combining sorfequiline, pretomanid, and linezolid (SPaL) demonstrated greater early bactericidal activity than standard isoniazid, rifampin, pyrazinamide, and ethambutol (i.e., HRZE), with higher-dose SPaL arms outperforming bedaquiline-containing comparators (52). Although peer-reviewed data are forthcoming, these findings provide a strong mechanistic rationale for upgrading ATP synthase inhibitors in future ultra-short regimens.

TBAJ-587 advances the diarylquinoline lineage further. Preclinical studies show higher potency, improved safety margins, and enhanced pharmacokinetics relative to bedaquiline (51). Now in Phase 1 evaluation, TBAJ-587 may ultimately serve as a safer, more potent ATP synthase inhibitor for incorporation into next-generation regimens.

### 5.2 Cytochrome bc1 inhibition: telacebec (Q203)

Telacebec (Q203) remains the leading cytochrome bc1 inhibitor in clinical development. By targeting the QcrB subunit, telacebec disrupts electron transport and the proton motive force required for ATP synthesis (28, 52). These effects complement ATP synthase inhibition and may enhance sterilizing activity. A Phase 2a early bactericidal activity trial demonstrated dose-dependent reductions in bacterial load among patients with drug-susceptible pulmonary TB, supporting its further development as a potential backbone for ultra-short regimens (52).

### 5.3 Dpre1 inhibition: BTZ-043 and benzothiazinones

Benzothiazinones such as BTZ-043 inhibit DprE1, an essential epimerase in arabinogalactan synthesis. Because DprE1 is located on the periplasmic side of the cell envelope, these compounds interact directly with the enzyme and maintain potency even in complex cell-wall environments (28, 53). A Phase 1b–2a trial in South Africa reported dose-proportional bactericidal activity and favorable tolerability, reinforcing BTZ-043’s potential as a cornerstone of future cell-wall–targeted regimens (53).

### 5.4 Pretomanid and the BPaL platform

Pretomanid continues to play a central role in BPaL and BPaLM regimens for highly drug-resistant TB. Through respiratory poisoning, nitric oxide release, and cell-wall lipid synthesis interference, pretomanid enhances sterilizing activity and supports substantial reductions in treatment duration (54). Implementation studies from the United States reported high treatment success when BPaL was paired with optimized linezolid dosing to reduce toxicity (55–57). As next-generation ATP synthase inhibitors such as TBAJ-876 (51–58) enter trials, pretomanid’s mechanistic complementarity positions it as a key component of future combinations.

### 5.5 Summary of therapeutic pipeline significance

Collectively, these mechanistically distinct agents—TBAJ-876, TBAJ-587, telacebec, BTZ-043, and pretomanid—represent a strategically aligned pipeline capable of producing shorter, safer, and more potent regimens. Their development reflects a deliberate focus on biochemical pathways essential for persistence, sterilization, and resistance suppression, reinforcing the field’s shift toward rational, mechanism-driven regimen design.

## 6 Integration of mechanistic advances into regimen design

Therapeutic discoveries reported in 2025 reinforce a central principle of tuberculosis regimen design; combining agents that target distinct but complementary biochemical pathways can maximize bactericidal and sterilizing activity. Mechanistic diversity not only improves efficacy across bacterial subpopulations—including replicating, slowly replicating, and metabolically constrained bacilli—but also reduces the probability of emergent drug resistance.

Next-generation combinations incorporating ATP synthase inhibitors (e.g., TBAJ-876), cytochrome bc1 inhibitors (e.g., telacebec), DprE1 inhibitors (e.g., BTZ-043), nitroimidazoles (e.g., pretomanid), and optimized oxazolidinones (e.g., linezolid or delpazolid) exemplify rational, mechanism-driven regimen design. Each class contributes unique strengths: ATP synthase inhibitors collapse cellular energy production; cytochrome bc1 inhibitors disrupt oxidative phosphorylation; benzothiazinones block essential cell-wall assembly; and nitroimidazoles provide potent activity against hypoxic or persistent bacilli. Regimens that simultaneously impair ATP synthesis, electron transport, and cell-wall biosynthesis are particularly promising because they attack vulnerabilities operative across physiological states (28, 42, 59).

By integrating drugs with complementary mechanisms, these regimens hold strong potential to shorten treatment, improve tolerability, and simplify monitoring requirements. Such strategies align with the 2030 global TB elimination goals by offering broader activity against diverse bacillary populations, reducing relapse risk, and enhancing treatment completion—critical attributes for achieving population-level impact. While these shorter regimens are promising, their programmatic scale-up will require careful attention to the potential risk of drug resistance, underscoring the need for parallel expansion of baseline resistance testing, pharmacovigilance, and surveillance systems.

## 7 Immunologic research and vaccine development in 2025

Vaccine development remains central to long-term TB elimination, and several candidates progressed in 2025. MTBVAC, a live-attenuated *M. tuberculosis* vaccine, demonstrated a favorable safety profile and elicited significantly stronger Th1-restricted CD4^+^ T-cell responses than BCG in a Phase 1b–2a trial (60). These findings, consistent with MTBVAC’s rational design that retains immunodominant antigens absent in BCG, support advancement to Phase 3 efficacy evaluation.

The M72/AS01_E_-4 vaccine similarly advanced, with a Phase 2 trial reporting durable antigen-specific T-cell and antibody responses in virally suppressed adolescents and adults living with HIV (61)—a population with disproportionate TB mortality. Although efficacy results are not yet available, enrollment of approximately 20,000 participants in the phase 3 M72/AS01E vaccine trial was completed in 2025, marking a major milestone toward generating definitive evidence on its protective effect against tuberculosis. Other vaccine candidates showed more modest results. H56:IC31 failed to reduce recurrence among adults who recently completed treatment despite strong immunogenicity (62), and a Phase 2b evaluation of BCG revaccination did not reduce sustained interferon-*γ* release assays conversion in adolescents (63). These outcomes underscore the continued uncertainty regarding immunologic correlates of protection.

New evidence in 2025 refined understanding of susceptibility and host variation in vaccine response. *The Lancet Seminar* emphasized that BCG vaccination may reduce both infection and disease, as BCG-vaccinated contacts are less likely to have positive interferon-*γ* release assays, suggesting partial protection against establishing infection itself (5). Age- and time-dependent analyses showed that the risk of progression is highest in young children and in the first two years after infection, reinforcing the importance of identifying vulnerable groups for next-generation vaccines.

Several immunologic discoveries provided further mechanistic insight. A Mendelian-based randomization study demonstrated that genetically reduced IL-6 signaling was associated with lower TB risk (64), challenging long-standing concerns that IL-6 pathway inhibition increases susceptibility and raises the possibility of targeted IL-6 modulation as a complementary immune strategy. Additionally, meta-analytic evidence confirmed that prior BCG vaccination reduces interferon-*γ* release assay conversion in exposed contacts (65), supporting the use of test conversion as a possible surrogate endpoint for early vaccine trials.

Advances in immunometabolism added another important dimension. TB infection induces strong upregulation of indoleamine 2,3-dioxygenase-1 (IDO1), shifting tryptophan metabolism toward kynurenine and generating an immunosuppressive granuloma microenvironment that restricts T-cell trafficking and reduces chemokine signaling (66). Plasma kynurenine-to-tryptophan ratios rise during active disease, fall with effective therapy, and correlate with severity, highlighting potential use as an immunologic biomarker for infection activity or treatment response (67). These findings expand understanding of how metabolic circuits shape TB immunity, may influence vaccine performance, and point to modifiable pathways that could enhance vaccine-elicited T-cell responses (27).

Collectively, these advances underscore both the promise and complexity of TB immunologic research and vaccine development. While several candidates induce strong immune responses, translating immunogenicity into durable protection will require deeper understanding of the metabolic, cytokine, and developmental factors that govern susceptibility and immunity. Accelerating vaccine development will depend on improved correlates of protection, robust endpoints, and the integration of immunometabolic insights into trial design.

## 8 Host-directed therapies and complementary mechanistic strategies

The limitations of pathogen-targeted therapy—including prolonged treatment duration, drug toxicity, and risk of relapse—continue to drive interest in host-directed therapies (HDTs) that modulate immune pathways, enhance intracellular drug delivery, or mitigate harmful inflammation. Several advances in 2025 strengthened the mechanistic basis for HDTs and clarified how they may complement antimicrobial regimens.

Macrophage-targeted nanoparticle delivery systems continued to show promise, enabling antimicrobials to reach intracellular compartments harboring *M. tuberculosis* and improving intralesional drug penetration (27). These technologies may facilitate regimen shortening when paired with potent antimicrobials targeting ATP synthase, cytochrome bc1, or DprE1.

Another major 2025 advance was the demonstration that glucocorticoids can directly reprogram human macrophage metabolism while preserving antimicrobial function. Dexamethasone markedly reduced basal and compensatory glycolysis in airway and monocyte-derived macrophages by downregulating the rate-limiting enzyme PFKFB3 (68). Despite broad suppression of cytokine secretion, dexamethasone enhanced intracellular control of *M. tuberculosis*, increasing macrophage survival, reducing caspase-3/7 activation, and significantly lowering bacterial burden at 120 h (69). Although glucocorticoids broadly suppress pro-inflammatory cytokines, recent 2025 data demonstrate that dexamethasone alters macrophage immunometabolism while still permitting host antimicrobial activity in *M. tuberculosis*-infected cells. This dissociation of anti-inflammatory and antimicrobial pathways expands understanding of host metabolic circuits in TB immunity and highlights potential for targeted or localized glucocorticoid delivery to balance inflammation and infection control.

Beyond glucocorticoids, additional HDT strategies under evaluation include modulation of cytokine pathways, mitochondrial metabolism, and autophagy. As mentioned above, a Mendelian-randomization analyses linking genetically reduced IL-6 signaling to decreased TB risk (69) further support selective immunomodulation as a potential pathway for therapeutic benefit. However, these genetic associations should not be interpreted as evidence that pharmacologic modulation of the IL-6 pathway is safe or effective for tuberculosis prevention or treatment.

Overall, HDT research remains early but increasingly mechanistically informed. The expanding understanding of host metabolic and inflammatory circuits—including glycolysis, phagolysosomal maturation, and cytokine regulation—provides a strong rationale for HDT-focused clinical trials that complement antimicrobial activity, reduce relapse risk, and improve long-term lung health.

## 9 Updating nomenclature, noumenon, and other nuances in 2025

Recent scholarship has challenged longstanding assumptions about how tuberculosis is conceptualized, diagnosed, and described across the disease spectrum. A 2025 analysis demonstrated that commonly used symptom-based dichotomies—such as “*asymptomatic*” vs. “*symptomatic*” TB or “*subclinical*” vs. “*clinical*” TB—are inadequate classifiers of disease state and lack both biological and operational validity for programmatic or research use (70). McCreesh and colleagues demonstrate that self-reported symptoms are an inadequate and unstable classifier of disease state, subject to interviewer variability, social context, recall, stigma, and unrelated comorbid conditions; nearly half of individuals with bacteriologically confirmed TB in prevalence surveys do not report symptoms at screening, and a sizeable proportion of “*TB symptoms*” in both TB and non-TB populations likely arise from unrelated causes. Adopting terms such as “*asymptomatic TB*” risks reifying an artificial conceptual boundary, misleadingly implying a distinct, milder condition, and inadvertently endorsing symptom-based screening—the very practice shown to miss a large share of TB cases and to lack empirical evidence for population-level impact (71). Together, these critiques highlight the need for TB nomenclature that reflects pathophysiology rather than presentation, and that avoids embedding clinical uncertainty into policy language. Both manuscripts emphasized that symptoms lie on a continuum, not a categorical boundary, and should not serve as the foundation for disease definitions, epidemiologic estimates, or treatment algorithms. Instead, they call for objective, reproducible indicators of disease severity—including bacteriologic burden, radiographic extent, and functional impairment—to guide research and programmatic strategies.

This evolution in TB conceptualization parallels advances in characterizing post-tuberculosis lung disease (PTLD), another area in which inconsistent terminology has hampered evidence synthesis. Recent reviews catalog a wide spectrum of persistent respiratory impairment, from airflow obstruction and fibrosis to chronic infectious complications, and emphasize the need for structured, standardized descriptors across clinical, radiologic, and functional domains (72). Building on this, a 2025 *Lancet Infectious Diseases* Personal View proposed the field’s first research case definition for PTLD, anchored in three domains—lung function, respiratory symptoms, and imaging abnormalities—while requiring attribution to prior TB and explicit exclusion of active disease (73). This definition was intentionally specific, feasible for high-burden settings, and accompanied by a transparent reporting framework to harmonize data across studies.

Collectively, these contributions signal a broader shift in TB science: away from symptom-derived labels and toward mechanistically grounded, empirically validated descriptors that capture the full continuum of TB-related pathology; from preclinical disease states without reported symptoms to chronic post-treatment sequelae. Symptom-based screening, while imperfect, remains operationally useful and will likely continue to complement newer diagnostic approaches during programmatic transition. Updating TB nomenclature in this direction has profound implications for surveillance, screening algorithms, targeted interventions, vaccine endpoint development, and the design and interpretation of diagnostic and therapeutic studies.

## 10 HIV–tuberculosis research in 2025

People with HIV (PWH) remain disproportionately affected by tuberculosis, motivating continued efforts to optimize tuberculosis prevention, treatment, and vaccine strategies in this population. Clinical trials published in 2025 addressed key challenges in HIV-associated TB, including pharmacologic compatibility between TB regimens and current antiretroviral therapy (ART) regimens, limitations in TPT delivery, and the need for safe and immunogenic vaccines for PWH. Together, these studies emphasize the importance of interventions that are biologically sound, ART-compatible, and feasible for implementation within integrated HIV programs (27, 74, 75).

Evidence informing TB treatment optimization in PWH during 2025 was derived largely from pharmacokinetic, safety, and subgroup analyses rather than HIV-specific efficacy trials. In the HARVEST trial of tuberculous meningitis, high-dose rifampicin did not improve six-month survival compared with standard dosing and was associated with a higher frequency of drug-induced liver injury, underscoring the need for caution when intensifying rifamycin exposure in this severe form of HIV-associated TB (75). Although not designed specifically for PWH, the TRUNCATE-TB trial evaluated multiple eight-week regimens for rifampicin-susceptible pulmonary TB and demonstrated the feasibility of regimen shortening using potent drug combinations, providing supportive evidence relevant to high HIV-prevalence settings where treatment simplification remains a priority (76).

TPT was a major focus of HIV-specific trials in 2025. The DOLPHIN-TOO study demonstrated that once-weekly rifapentine and isoniazid can be initiated concurrently with dolutegravir-based ART in ART-naïve PWH without clinically meaningful safety concerns or loss of antiretroviral drug exposure, supporting streamlined co-initiation strategies (77). In contrast, the CAT cluster-randomized trial showed that a low-intensity, behavioral intervention designed to influence prescribing choices (choice architecture) did not consistently increase TPT prescribing at ART initiation across countries, highlighting persistent implementation barriers that extend beyond regimen selection alone (78).

Immunologic investigations in 2025 focused primarily on vaccine evaluation rather than treatment response. A Phase 2 trial of the investigational M72/AS01_E_-4 vaccine in PWH demonstrated an acceptable safety profile and robust humoral and CD4^+^ T-cell responses, supporting inclusion of PWH in ongoing late-phase efficacy trials (61). These findings address a critical evidence gap by confirming that novel TB vaccines can be safely administered and are immunogenic in virally suppressed PWH.

Pharmacokinetic studies further informed TB management in the context of ART. The ANRS12292-RIFAVIRENZ sub-study demonstrated substantial interindividual variability in rifampicin and isoniazid exposure among PWH receiving efavirenz-based ART, with efavirenz reducing rifampicin exposure and higher rifampicin dosing markedly improving attainment of target concentrations. These findings provide mechanistic support for dose optimization strategies in people with HIV and TB, particularly where efavirenz-based regimens remain in use (79).

In 2025, long-acting antiretroviral agents for HIV treatment and prevention were extensively studied and increasingly positioned for broader implementation (80–83). As these agents advance, tuberculosis diagnosis, treatment, and prevention must be explicitly considered in HIV/TB co-endemic settings. Long-acting antiretroviral exposure may be substantially affected by CYP3A4 induction and inhibition, particularly with rifamycin-based regimens used for TB treatment and TPT. In parallel, bedaquiline and several antiretroviral agents share CYP3A4-mediated disposition, creating bidirectional drug–drug interaction concerns (84, 85). Co-administration may result in subtherapeutic antiretroviral exposure with implications for treatment efficacy, prevention effectiveness, and selection of resistance (85). These challenges are compounded by the frequent exclusion of individuals receiving TB treatment from major long-acting pre-exposure prophylaxis trials (81, 82), despite TB remaining a leading cause of mortality among PWH globally (1, 86). Ensuring pharmacologic compatibility between antiretroviral and TB regimens, strengthening pharmacovigilance, and evaluating co-formulated or staggered delivery strategies for TB treatment and TPT alongside long-acting antiretrovirals will be essential for effective programmatic integration. Addressing these considerations is important to overcoming implementation challenges highlighted in this review, including improving access to TB prevention and care, and to advancing progress toward End TB Strategy milestones (4, 86–88).

Taken together, HIV/TB trials published in 2025 reinforce the importance of ART-compatible TPT, cautious optimization of rifamycin dosing, and continued inclusion of PWH in TB vaccine development. While gaps remain—particularly regarding HIV-specific efficacy data for ultra-short TB treatment regimens and integrating TPT/ATT into long-term uses of injectable long-acting antiretrovirals—these studies provide a rigorous empirical foundation for integrated HIV-TB strategies aimed at reducing mortality and improving long-term outcomes among PWH.

## 11 Policy, financing, and implementation realities of 2025

Despite substantial scientific progress, structural and financial constraints continued to impede movement toward TB elimination in 2025. The WHO Global TB Report documented persistent gaps in case detection, suboptimal treatment success for drug-resistant TB, and chronic underfinancing of national programs, with global funding still far below the estimated US $22 billion needed annually to scale essential services (1, 3).

Implementation of new technologies remains limited by health-system capacity. Many high-burden settings lack laboratory infrastructure for molecular diagnostics and targeted next-generation sequencing, digital systems to support AI-assisted chest radiography, and procurement mechanisms to ensure stable access to newer medicines such as bedaquiline, pretomanid, and linezolid (9, 47). Workforce shortages and constrained regulatory pathways further delay adoption of updated guideline-recommended regimens (41, 86).

Concerns about unequal access are increasingly urgent. Without dedicated investment, advanced diagnostics and therapeutics risk diffusing primarily into high-income or better-resourced middle-income countries, widening disparities in TB incidence and outcomes—a trend repeatedly identified as a threat to global progress (1, 3, 86–89).

Transitions in the global health landscape in 2025, including uncertainty related to programmatic funding and prioritization, affected the pace of implementation of new TB tools in some settings (90, 91). Yet, the growing alignment of TB programs with pandemic preparedness and global health security agendas may offer opportunities for integrated investments in genomic surveillance, digital screening, and vaccine infrastructure (54, 60, 86–89).

## 12 Conclusion

In 2025, advances in diagnostics, therapeutics, vaccines, and host-directed strategies collectively strengthened the scientific foundation for TB elimination, while conceptual progress refined how TB disease is defined and measured. Yet these gains will only translate into population-level impact if matched by investments in implementation, increased access, and health-system capacity. Aligning innovation with delivery remains the central challenge—and the central opportunity—for accelerating global TB elimination.

As the late Dr. Paul Farmer consistently argued, “*tuberculosis is not only a biomedical problem but a political and moral one, such that scientific advances will translate into population-level impact only if matched by sustained commitments to equity, strong health systems, and the social conditions required to deliver care*” (92).

## Figures and Tables

**TABLE 1:** Mechanistic landscape of TB drug targets.

Category	Target	Class	Examples	Mechanism	Notes
Cell-wall biosynthesis	Mycolic-acid synthesis (*inh*A)	Isoniazid, thioamides; direct inhA inhibitors	Isoniazid, ethionamide; GSK3036656 (GSK656)	Inhibit inhA, blocking mycolic-acid synthesis	GSK656 = direct InhA inhibitor in clinical development
	Arabinogalactan synthesis (*dpr*El)	Benzothiazinones	BTZ-043, PBTZ-169 (macozinone)	Inhibits *dprEl* epimerase	Advanced clinical candidates
	Arabinogalactan synthesis *(dprE1)*	Non-covalent DprE1 inhibitors	OPC-167832, TBA-7371	Inhibit *dpr*El (non-covalent binding)	Improved safety/resistance profiles (clinical-stage)
	Arabinosyl transferases (emb)	Ethambutol	Ethambutol	Inhibits cell wall arabinan synthesis	First-line drug
Energy metabolism/oxidative phosphorylation	ATP synthase (c-subunit)	Diarylquinolines	Bedaquiline	Collapses ATP production	Backbone of modern MDR regimens
	ATP synthase (next-gen)	Diarylquinoline derivatives	TBAJ-876, TBAJ-587	Improved ATP synthase inhibition	Designed for less toxicity than bedaquiline
	Respiratory chain (cytochrome bc1)	Imidazopyridines	Q203 (telacebec)	Inhibits electron transport chain	Late-stage clinical development
	Respiratory chain (cytochrome bcc-aa3)	Phenoxyalkylbenzimidazoles	PAB compounds (e.g., PAB-1 series)	Disrupt oxidative phosphorylation	Preclinical/early pipeline
Protein synthesis	50S ribosomal subunit (23S rRNA)	Oxazolidinones	Linezolid, tedizolid	Inhibit peptidyl transferase activity	Established MDR/XDR TB drugs
	50S ribosomal subunit (next-gen oxazolidinones)	Oxazolidinones	Sutezolid, delpazolid, TBI-223	Same target, improved safety	Reduced mitochondrial toxicity vs. linezolid
	30S ribosomal subunit	Aminoglycosides	Amikacin, streptomycin	Inhibit protein synthesis	Second-line injectable agents
Nucleic-acid synthesis	DNA gyrase	Fluoroquinolones	Moxifloxacin, levofloxacin	Inhibit DNA replication	Core second-line drugs
	DNA gyrase (next-gen)	Novel gyrase inhibitors	SPR720 (prodrug of SPR719)	Gyrase inhibition (distinct binding)	Active vs. resistant strains (clinical dev.)
	RNA polymerase	Rifamycins	Rifampin, rifabutin, rifapentine	Inhibit RNA synthesis	First-line backbone
Other pathways	Prodrug activation/redox	Nitroimidazoles	Pretomanid, delamanid	Generate reactive nitrogen species	Key in BPaL regimen
	Coenzyme A biosynthesis	Pantothenate pathway inhibitors	SQ109 (multi-target)	Disrupts membrane & energy metabolism	Multi-mechanistic; clinical-stage
	Cholesterol metabolism	Host/pathogen lipid metabolism	Statin adjuncts (host-directed)	Alter host lipid pathways	Adjunctive therapy approach
Host-directed therapy	Immune modulation	Various (e.g., mTOR inhibitors, NSAIDs)	Metformin, everolimus (investigational)	Enhance host response	Adjunct strategies under study
